# Clinical features, radiological profiles, pathological features and surgical outcomes of pituicytomas: a report of 11 cases and a pooled analysis of individual patient data

**DOI:** 10.1186/s40779-021-00332-5

**Published:** 2021-07-02

**Authors:** Jian-Hua Cheng, Ding Nie, Bin Li, Song-Bai Gui, Chu-Zhong Li, Ya-Zhuo Zhang, Luigi Maria Cavallo, Peng Zhao

**Affiliations:** 1 Neurosurgical Department, Beijing Tiantan Hospital, Capital Medical University, No. 119 South Fourth Ring West Road, Fengtai District, Beijing, 100070 China; 2 Department of Cell and Biology, Beijing Neurosurgical Institute, Beijing, 100070 China; 3 Division of Neurosurgery, Universitá Degli Studi di Napoli Federico II, 80142 Naples, Italy

**Keywords:** Pituicytoma, Endoscopic transsphenoidal surgery, Craniotomy, Posterior pituitary lobe tumor

## Abstract

**Background:**

Pituicytoma is an extremely rare low-grade glial tumor that is closely related to the neurohypophysis axis. Most studies of pituicytomas include only several cases. To better understand this disease, we reviewed a series of cases of pituicytomas. The diagnosis and treatment of pituicytoma must be further elucidated.

**Methods:**

Eleven patients with pituicytoma admitted to Beijing Tiantan Hospital from 2012 to 2019 were selected. The clinical features, including radiological and histological examination, surgical records and prognosis were reviewed. Sixty-eight other previously published cases of pituicytoma also were used to analyze the predictive factors for the results. The Cox regression model was used for univariate and multivariate analyses.

**Results:**

Our patients included 5 males (45.5%) and 6 females (54.5%), with a mean age of 49.3 years. The tumor was located in the suprasellar region in 5 patients (45.5%), intrasellar region in 4 patients (36.4%), and intrasellar-suprasellar region in 2 patients (18.2%). All patients were misdiagnosed with other common tumors in the sellar region before the operation. During the operation, gross total resection (GTR) of the tumor was achieved in 6 patients (54.5%), and subtotal resection (STR) was achieved in 5 patients (45.5%). The mean progression-free survival (PFS) time was 29.82 months. Tumor progression after surgical resection occurred in 4 patients (36.4%). Among them, 60.0% of the patients (cases 4, 5, 7) with STR experienced progression, while 16.7% of the patients (case 2) with GTR experienced progression. Combined with the 68 cases in the literature, GTR was an independent risk factor for PFS time (*P* < 0.05).

**Conclusions:**

Pituicytomas are more common in middle-aged people and the sellar region. The clinical manifestations of pituicytomas are different, but no diagnostic clinical features have been identified other than an abnormally abundant blood supply. Currently, GTR is the best approach for the treatment of pituicytomas. More patients and longer follow-up periods were needed to further elucidate the biological features of pituicytomas.

**Supplementary Information:**

The online version contains supplementary material available at 10.1186/s40779-021-00332-5.

## Background

Pituicytoma is a benign fusiform astrocytoma that originates from the neurohypophysis or pituitary stalk [1–3]. It is a localized, generally solid, low-grade glial tumor that consists of bipolar fusiform cells arranged in bundles or striations [4]. Pituicytomas are exceedingly rare. Currently, approximately 119 cases of pituicytoma have been reported [5, 6].

Pituicytoma is easily confused with other tumors in the sellar region. However, it has been differentiated from other tumors in the sellar region based on microscopic cell morphology and immunohistochemistry [7]. The treatment of pituicytoma generally involves surgical resection [8]. Here, this study reports 11 patients with pathologically proven pituicytoma and their long-term follow-up results. To the best of our knowledge, this study involves the largest number of patients with pituicytoma from a single center. We believe that the clinical, radiological, and pathological features of pituicytomas studied here will facilitate the better diagnosis and treatment of pituicytomas in the future.

## Methods

### Clinical data

This study retrospectively analyzed the clinical features and surgical results of patients with histologically diagnosed pituicytoma who were treated at Beijing Tiantan Hospital from 2012 to 2019. This study was approved by the Ethics Committee of Beijing Tiantan Hospital (KYSQ2019–228-01). All patients underwent cranial contrast magnetic resonance imaging (MRI) and testing of their hormone levels before the operation. The clinical data were collected by reviewing clinical case information and telephone follow-up and included clinical symptoms, radiographic examinations, surgical results, immunohistochemical results, and follow-up results.

### Surgical treatment

The preoperative examination was completed, and contraindications were excluded. All patients underwent endoscopic transsphenoidal surgery (ETS) or craniotomy under general anesthesia. The extent of tumor resection was divided into gross total resection (GTR) and subtotal resection (STR) based on an analysis of the postoperative cranial contrast MRI and surgical records. Mucosal flap repair was performed in all patients with definite or suspected cerebrospinal fluid leakage during ETS.

### Pathological examination

The tumor specimens of all patients were sent for a pathological evaluation, including hematoxylin and eosin (HE) staining and immunohistochemical staining.

Postoperative immunohistochemical staining was performed with antibodies against glial fibrillary acidic protein (GFAP, Abcam, UK), S100 (Abcam, UK), vimentin (Abcam, UK), thyroid transcription factor-1 (TTF-1, Abcam, UK), endomysial antibody (EMA, Abcam, UK), CD34 (Abcam, UK), MIB-1 (Abcam, UK), progesterone receptor (PR, Abcam, UK), smooth muscle actin (SMA, Abcam, UK). Pituicytomas were diffusely positive for the S100 and vimentin proteins, weakly to moderately positive for GFAP, and displayed nuclear immunoreactivity for TTF-1 and a punctate distribution of EMA-positive staining in the cytoplasm [8].

### Literature research

**Fig. 1:**
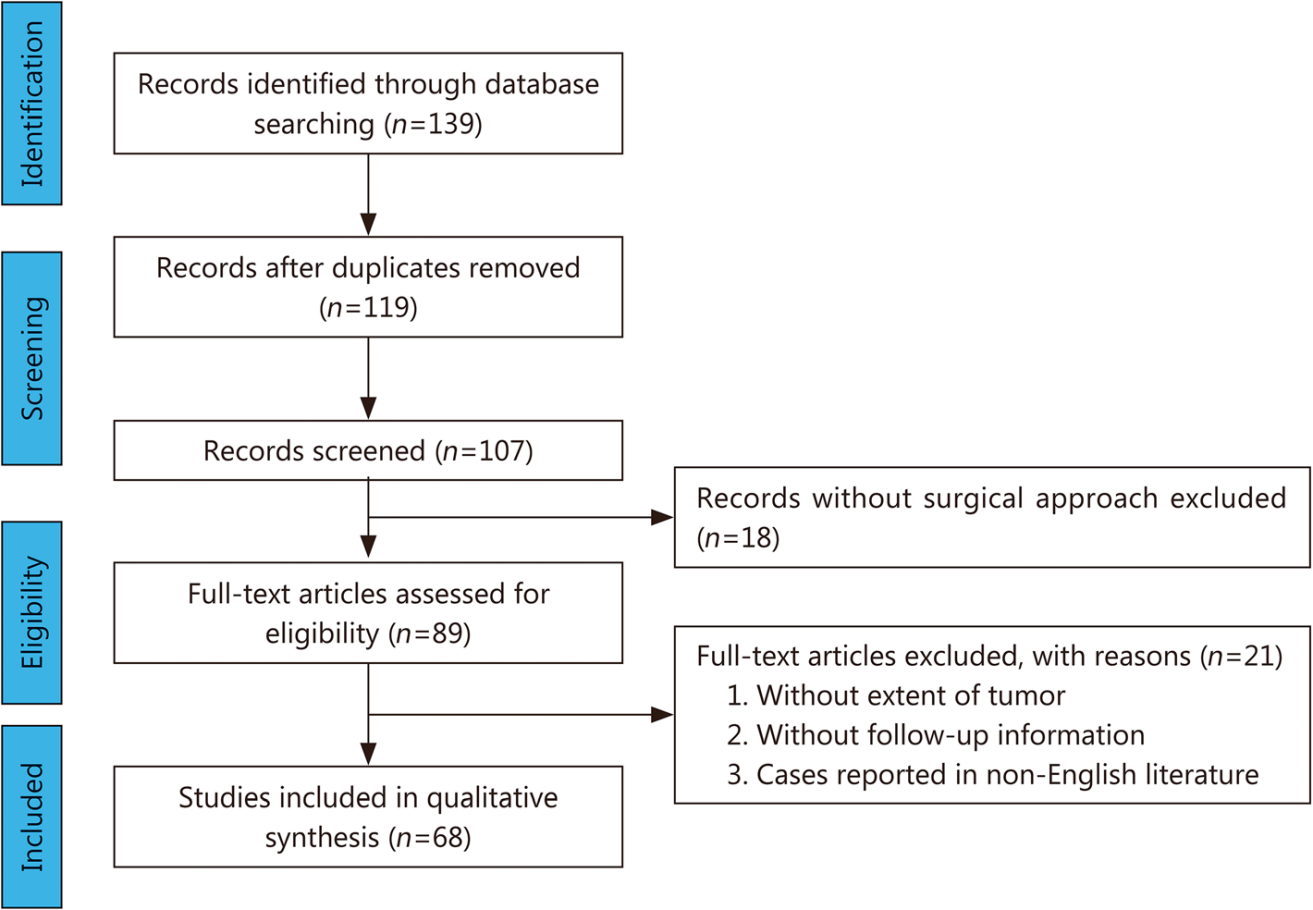
PRISMA flow-chart

The literature on pituicytoma published from January 1958 to January 2020 was searched on PubMed using the keyword “pituicytoma”. The following selection criteria were used: (1) patients with pituicytoma confirmed by an operation and pathology; (2) patients who had been followed for more than half a year; and (3) cases reported in English literature. The exclusion criteria were as follows: (1) patients with pituicytoma and other intracranial tumors; (2) patients with other space-occupying lesions in the sellar region. The information from each patient was carefully reviewed by two authors (JHC and DN) and any repeated patients were excluded. The information collected included the general condition of the patients (sex, age at the time of diagnosis, and location of the tumor), the treatment (surgical approach, extent of tumor resection, radiotherapy and chemotherapy), and outcome (tumor recurrence or progression). All the steps of the process followed the PRISMA flow diagram as presented in Fig. 1. Sixty-eight cases of pituicytoma reported in the literature are listed in Supplementary Table 1.

### Follow-up

The clinical data analyzed in this study, including clinical symptoms, radiographic examination, surgical results, immunohistochemical results, and follow-up results, were collected by reviewing clinical case information and telephone follow-up. Among these parameters, the clinical history of the patients was collected by a retrospective chart review. Potential risk factors assessed here included age, sex, tumor size and location, surgical resection extent, and other adverse predictors, such as progression-free survival (PFS).

### Statistical analysis

The Cox regression model was used for univariate and multivariate analyses with the IBM SPSS statistics software package (version 22.0 IBM Corp). Significance was set to *P* < 0.05 in all analyses.

## Results

### Patient’s clinical features

Eleven patients with pituicytoma in our hospital were included in this study. Table 1 records the clinical features and treatment of the patients. Pituicytoma occurred in 5 males (45.5%) and 6 females (54.5%), aged from 32 to 65 years, with an average age of 49.3 years. Preoperative symptoms included vision loss (3/11, 27.3%), headache (3/11, 27.3%), dizziness (2/11, 18.2%), endocrine disorders (1/11, 9.1%), and a lack of symptoms (2/11, 18.2%). Among all patients, 63.6% (7/11) were undergoing their first operation for pituicytoma, while 36.4% (4/11) were undergoing surgery due to recurrence. Notably, case 5 was diagnosed as pituitary adenoma by pathology in the first operation, while the pathological diagnosis obtained in the second operation was pituicytoma.

**Table 1:** Clinical, radiologic, and pathological characteristics of 11 cases with pituicytomas

No.	Sex	Age (year)	Signs and symptoms	Tumor location	Surgical approach	Extent of resection	Radiology	Postoperative complications	Immuno-histochemistry	PFS (mo)	Recurrence
1	M	46	Dizzy	Suprasellar	Craniotomy	GTR	Solid	Hypophysis, intracranial infection	S100(+), EMA(+), TTF-1(+), Ki-67 (2%)	12	No
2	F	46	None	Suprasellar	Craniotomy	GTR	Solid	Hypophysis	S100(+), GFAP(+), TTF-1(+), EMA(+), CD34(+), Ki-67 (1%)	34	Yes
3	F	47	Headache	Intrasellar	ETS	GTR	Solid	Hypophysis, intracranial infection	GFAP(+), TTF-1(+), CD34(+), S100(+), PR(+), EMA(+), Ki-67 (1%)	34	No
4	M	63	Vision loss	Intrasellar and suprasellar	ETS	STR	Uneven softness and toughness	None	S100(+), GFAP(+), TTF-1(+), EMA(+), Ki-67 (1%)	36	Yes
5	M	53	Vision loss	Intrasellar	Craniotomy	STR	Solid	None	S100(+), GFAP(+), TTF-1(+), EMA(+), vimentin(+), Ki-67 (1%)	88	Yes
6	M	32	Headache with decreased vision	Suprasellar	Craniotomy	GTR	Solid	Hypophysis	GFAP(+), S100(+), EMA(+), TTF-1(+), Ki-67 (1–3%)	24	No
7	F	51	Vision loss	Intrasellar	Craniotomy	STR	Uneven softness and toughness	Hypophysis	GFAP(+), S100(+), EMA(+), TTF-1(+), Ki-67 (1–5%)	22	Yes
8	F	65	None	Intrasellar and suprasellar	Craniotomy	STR	Soft	None	S100(+), GFAP(+), TTF-1(+), EMA(+), Ki-67 (1%)	12	No
9	F	38	Menstrual disorder	Suprasellar	Craniotomy	STR	Solid	None	GFAP(+), S100(+), EMA(+), TTF-1(+), CD34(+), Ki-67 (4%)	28	No
10	F	54	Headache	Intrasellar	ETS	GTR	Soft	None	S100(+), EMA(+), TTF-1(+), CD34(+), SMA(+), Ki-67 (1–3%)	22	No
11	M	47	Dizzy	Suprasellar	Craniotomy	GTR	Solid	Hypophysis and anemia	S100(+), EMA(+), TTF-1(+), Ki67 (2%)	16	No

*M* Male, *GTR* Gross total resection, *EMA* Endomysial antibody, *TTF-1* Thyroid transcription factor-1, *F* Female, *GFAP* Glial fibrillary acidic protein, *PR* Progesterone receptor, *ETS* Endoscopic transsphenoidal surgery, *STR* Subtotal resection, *SMA* Smooth muscle actin

### Patient’s imaging characteristics

**Fig. 2:**
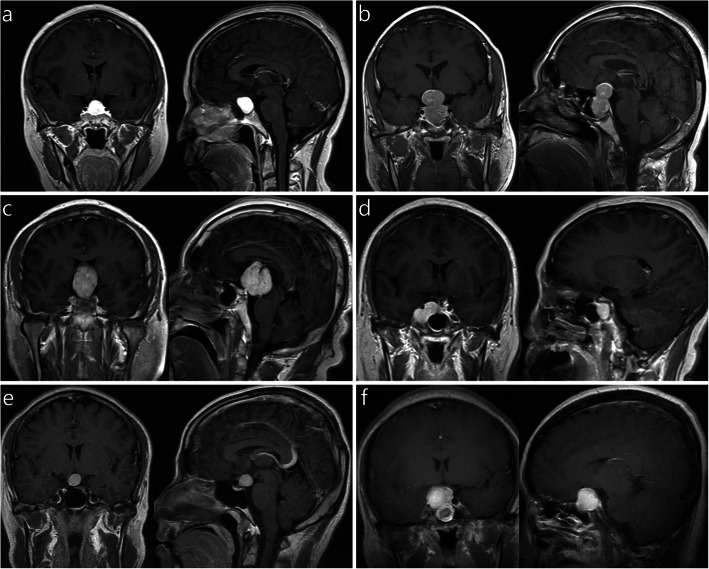
Radiological features of pituicytomas. **a.** The tumor is completely located in the intrasellar region. **b.** The tumor is located in the intrasellar-suprasellar region. **c.** The tumor is completely located in the suprasellar region. **d.** Pituicytoma invading the right cavernous sinus. The lesion surrounded the internal carotid artery, and the optic chiasm was not compressed. **e.** The optic chiasm was oppressed and elevated by the tumor, and the cavernous sinus was not involved. **f.** Pituicytoma invading the right cavernous sinus; the optic chiasm was compressed, and the tumor was accompanied by cystic degeneration

Suprasellar lesions were diagnosed in 5 patients (45.5%), intrasellar lesions in 4 patients (36.4%), and intrasellar-suprasellar lesions in 2 patients (18.2%) (Fig. 2a-c, Table 1). MRI showed a clear boundary of the tumor in 10 patients (90.9%) and an unclear boundary in 1 patient (9.1%). Contrast-enhanced MRI showed homogeneous enhancement in 6 patients (54.5%), inhomogeneous enhancement in 5 patients (45.5%), and cystic degeneration in 3 patients (27.3%). Obvious vascular flow emptiness was observed in 1 patient (Fig. 2c). The parenchyma of the tumor showed isointense T_1_ and T_2_ signals in 7 patients (63.6%), hyperintense T_1_ and hyperintense T_2_ signals in 2 patients (18.2%), and isointense T_1_ and hyperintense T_2_ signals in 2 patients (18.2%). The maximum tumor diameter ranged from 15 to 32 mm, with a mean size of 24.2 mm. The tumor size was 10–30 mm in 9 patients (81.8%) and larger than 30 mm in 2 patients (18.2%). The tumor compressed the optic chiasm in 7 patients (63.6%) (Fig. 2d), and invaded the cavernous sinus or internal carotid artery in 4 patients (36.4%) (Fig. 2e). The tumor both oppressed the optic chiasm and invaded the cavernous sinus or internal carotid artery in 2 patients (18.2%) (Fig. 2f).

In our study, all patients were misdiagnosed before surgery. Four patients (36.4%) were misdiagnosed with craniopharyngioma, and 7 patients (63.6%) were misdiagnosed with pituitary adenoma.

### Surgical treatments and outcomes

**Fig. 3:**
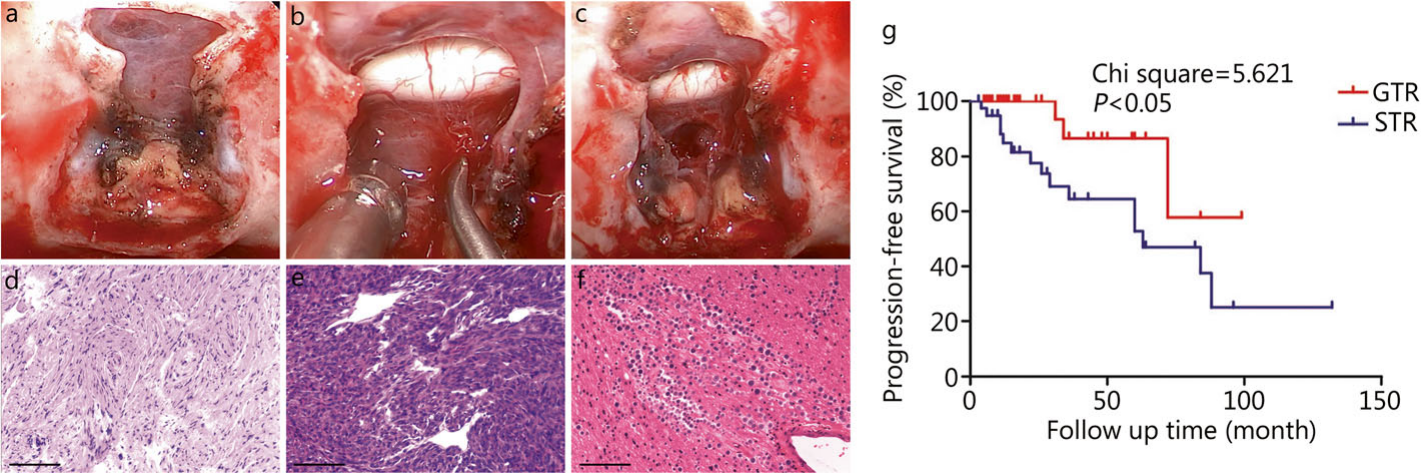
Endoscopic transsphenoidal surgery to remove a pituicytoma. **a.** The saddle bottom was successfully exposed, and the bone structure of the saddle bottom was smoothed. **b.** Opening the bottom of the saddle to expose the tumor. The tumor is red, the blood supply is rich, and it is adjacent to the optic nerve. **c.** The tumor was successfully removed, and the optic nerve was well protected. **d-f.** Histopathological examination of the pituicytoma specimen. **g.** Kaplan-Meier survival curve showing the effects of tumor gross total resection (GTR) and subtotal resection (STR) on progression-free survival (PFS) in patients (Chi square = 5.621, *P* < 0.05). All scale bars = 100 μm

All patients underwent surgical resection, including 8 patients (72.7%) who underwent a craniotomy and 3 patients (27.3%) who underwent ETS. The standard transsphenoidal approach was used in 2 patients (18.2%), and the extended transsphenoidal approach was used in 1 patient (9.1%). GTR was performed in 6 patients (54.5%), and STR was performed in 5 patients (45.5%). During the operation, the tumor was hard in 7 patients (63.6%), soft in 2 patients (18.2%), and uneven in 2 patients (18.2%). All tumors were red or grayish red and the blood supply was extremely abundant; 2 patients (18.2%) received an intraoperative blood transfusion because of excessive bleeding. Postoperative complications of the craniotomy included hypopituitarism in 5 patients (62.5%) and intracranial infection in 1 patient (12.5%). In our cases, 5 patients (45.5%) had diabetes insipidus due to hypophysis after the operation. The average postoperative hospital stay of patients undergoing craniotomy was 18.4 (range: 8–48) days. The average postoperative recovery time of patients undergoing ETS was 8.2 (range: 6–10) days. Figure 3a-c shows the resection of tumors during ETS.

After long-term follow-up, no patient received radiotherapy or chemotherapy after the operation. Recurrence occurred in 4 patients (36.4%) after the operation, with an average recurrence time of 48.6 months. The patients with postoperative recurrence received surgical treatment again at our hospital.

We further analyzed the factors affecting tumor progression. The average follow-up time for the whole group was 34.6 months. Four patients (36.4%) experienced tumor progression or recurrence, and the average PFS was 45 months. Overall, three patients with STR (60%) experienced progression, while one patient with GTR (16.7%) experienced progression.

### Results of immunohistochemical staining

After HE staining, the tumor cells were spindle-shaped, and the nuclei were deeply stained under a light microscope (Fig. 3d-f). The immunohistochemical results showed that all patients were S100-positive and EMA-positive, 8 patients (72.7%) were GFAP-positive, 4 patients (36.4%) were CD34-positive, and 1 patient (9.1%) was vimentin-positive. Positive TTF-1 expression was detected in all 11 patients using immunohistochemical staining. The Ki-67 proliferation index ranged from 1 to 3% in 9 patients (81.8%) and was greater than 3% in 2 patients (18.2%).

### Characteristics of pooled cases and long-term outcomes

After literature search, we collected the information from another 68 patients with complete data and follow-up data who were diagnosed with pituicytoma. In addition to our 11 patients, the prognosis of 79 patients was analyzed. Overall, the average age of the patients was 47.7 years, and the median age was 40.0 years; 39 patients (49.4%) were males and 40 patients (50.6%) were females. Thirty-eight patients (48.1%) were diagnosed with suprasellar tumors, 23 patients (29.1%) were diagnosed with intrasellar-suprasellar tumors, and 18 patients (22.8%) were diagnosed with intrasellar tumors. We further analyzed the factors affecting tumor progression. The average follow-up time for the whole group was 29.32 months (median follow-up time: 24 months). Nineteen patients (24.1%) experienced tumor progression or recurrence, and the average PFS was 35.73 months. The multivariate analysis showed that extent of resection was an independent risk factor for PFS (*HR* = 0.315, 95% CI 0.121–0.819, *P* < 0.05) (Table 2). The Kaplan-Meier analysis showed that the extent of tumor resection affected the PFS of patients (Fig. 3g).

**Table 2:** Univariate and multivariate analysis prognostic factors in the pooled 79 cases

Variable	Univariate analysis		Multivariate analysis	
	*HR* (95% CI)	*P*-value	*HR* (95% CI)	*P*-value
Age > 40 years	0.836 (0.273–2.569)	0.763		
Male	0.267 (0.095–0.750)	0.007	3.169 (0.809–12.259)	0.095
Extent of resection	0.282 (0.081–0.991)	0.027	0.315 (0.121–0.819)	0.047
Tumor location	1.645 (0.643–4.214)	0.301		
Surgical approach	1.098 (0.411–2.879)	0.857		

A subsequent analysis of the effect of the surgical approach on GTR of the tumor found that the craniotomy approach to achieve GTR accounted for 40% and the transsphenoidal approach to achieve GTR accounted for 46%. The total resection rates of intrasellar, intrasellar-suprasellar, and suprasellar tumors were 68.35, 30.38, and 50.63%, respectively.

## Discussion

Pituicytoma is extremely rare and has a high misdiagnosis rate. Compared with pituitary adenoma and craniopharyngioma, pituicytoma is an antecedent. It is not possible to diagnose pituicytoma pre-operatively, however a degree of suspicion should be held in large sellar lesions certain features on imaging. However, the statistical analysis of a large sample may enable the preoperative diagnosis of pituicytoma and choice a more suitable surgical approach, as well as better preparation for difficult intraoperative bleeding.

### Epidemiology and clinical characteristics

Pituicytoma, which is a low-grade neurogenic tumor (WHO I), is a very rare spindle-shaped astrocytic tumor that originates from the posterior lobe or stalk of the pituitary gland [1, 9]. To date, only 4 articles have reported more than 6 cases of pituicytoma [7, 10–12]. Thus, useful, detailed, and accurate information regarding the diagnosis and treatment of pituicytomas is difficult to obtain. The number of cases reported in this study is currently the largest number reported by a single center. From the case data we reported, we did not observe a significant gender difference, which was slightly different from the larger number of male patients reported in a previous study [6]. Pituicytomas can occur at any age but are more common in middle-aged and elderly individuals [8]. In our study, the average age of onset of patients was 49.3 years (range: 32–65 years). The clinical symptoms of pituicytomas vary due to the different sizes and locations of compression. Some of the adenopituitary insufficiency observed in the patients in this study without diabetes insipidus was due to the gradual, dorsal and nondestructive extension of the tumor at the lowest part of the pituitary stalk, which may cause vasopressin to leak into the systemic circulation before reaching the affected segment of the pituitary stalk, while the hypothalamic release of factors through the pituitary portal system is interrupted and tumor growth oppresses the optic nerve, resulting in visual field damage and pituitary compression. Similarly, tumor compression may cause hypopituitarism and headaches, and compression of the hypothalamus potentially causes mental symptoms [6, 8, 13]. Some patients also experience hyperprolactinemia due to secondary hyperprolactinemia caused by hypothalamic dopamine transport disorders induced by funnel compression [12], which was observed in patient 4, who had slightly increased prolactin levels before the operation. Most of the reported pituicytomas have clear boundaries, and a few have been reported to invade the cavernous sinus [14, 15].

### Radiological features

Pituicytomas are easily confused with other tumors in the sellar region, such as pituitary adenomas and craniopharyngiomas. Pituicytomas are mostly located in the suprasellar region. However, some are located in the sellar or the sellar-suprasellar region. Most pituicytomas are uniformly enhanced, some are inhomogeneous, and some cystic degeneration has been observed [16]. MRI showed that the tumors were solid masses, and most of them had clear boundaries. It has been reported in the literature that pituicytomas are isointense on T_1_-weighted images and slightly hyperintense or isointense on T_2_-weighted images [10]. In our patients, most pituicytomas showed isosignals on T_1_-weighted images and slightly hyperintense or isosignals on T_2_-weighted images. Some patients showed hyperintensity on T_1_-weighted and T_2_-weighted images. Some authors suggest that the early contrast enhancement of dynamic MRI or angiography should be used as a differential feature, which reflects the high vascularity of pituicytoma [17, 18]. The empty shadow of vascular flow was observed on T_1_-weighted images from patient 1, indicating the abundant blood supply of the tumor. Although pituicytoma has no special imaging features, it displays no calcification on CT and can be distinguished from craniopharyngioma. Pituitary adenomas are homogeneously enhanced and easily invade the surrounding tissues, which enable them to be distinguished from pituicytomas. Pituicytomas are distinguished from other tumors in the sellar region based on these radiological characteristics. Notably, pituicytoma lacks calcification and rarely exhibits cystic changes, which help to distinguish it from ameloblastic craniopharyngiomas in the sellar region or suprasellar region.

### Pathological findings

The final diagnosis of pituicytoma depends on the pathological findings. During the operation, the boundary of the pituicytoma is clear, and most tumors are tough and smooth. A few reports have described a soft texture or cystic degeneration [7, 19]. Most of the tumors are red or grayish red, with an abundant blood supply and no invasion of the surrounding tissue. The adhesion between the tumor originating from the pituitary stalk and the posterior pituitary lobe can be difficult to distinguish [18]. Immunohistochemical staining showed that S-100 was strongly positive [20], GFAP was invisible or weakly positive [7, 21], EMA was rarely positive, and TTF-1 positivity was helpful for the diagnosis of pituicytoma [8]. In our 11 patients, S-100 immunoreactivity was strongly positive, consistent with the diagnosis of pituicytoma.

According to Brat et al. [7], the diagnostic criteria for pituicytoma are spindle cell tumors, no or trace levels of nuclear atypia and mitotic phase. Immunohistochemical staining showed GFAP(+), S100(+) and vimentin(+). Because pituicytomas originate from pituitary cells, TTF-1 has been widely used in the diagnosis of pituicytomas in recent years [8]. Pituitary spindle cell oncocytoma and granulosa cell tumors are derivatives of pituicytoma. TTF-1 is also positive in spindle cell oncocytoma and granulosa cell tumors [22]. The neurohypophysis is a periventricular organ. Interestingly, the endplate vascular organ in the anterior part of the third ventricle also expresses TTF-1. Chordate gliomas located in this region also express TTF-1 [23]. The expression of TTF-1 in pituitary cytomas and chordate gliomas prompted the hypothesis that these tumors may belong to a series of lineage-related tumors in the basal forebrain [24]. In general, all pituicytomas showed diffuse nuclear labeling for TTF-1 and positive expression of EMA, S100 and GFAP to varying degrees. These findings were similar to other reported cases of pituicytomas [11].

### Treatments and outcomes

Currently, the pathogenesis of pituicytoma is still unknown. These tumors theoretically display benign biological behaviors with neither malignant histological features nor tumor-derived metastasis. Currently, the GTR of tumors is the primary treatment. The surgical approach includes transfrontotemporal craniotomy and a transnasal transsphenoidal approach. Compared with craniotomy, transsphenoidal surgery is minimally invasive and may reduce the risk of postoperative complications. The transsphenoidal approach includes the standard transsphenoidal approach and extended transsphenoidal approach. For giant pituicytomas invading the cavernous sinus, the extended transsphenoidal approach is adopted to achieve total resection. Most of the patients with poor preoperative vision experience varying degrees of clinical improvement after operation, and patients with poor vision undergo a craniotomy. However, the effect of the surgical approach on the GTR rate of pituicytomas must be further evaluated. Due to the rich blood supply of the tumor, uncontrollable bleeding is often encountered in the process of tumor resection, which leads to subtotal resection of the tumor [25–27]. However, angiographic studies are inevitably needed before a reoperation, and another goal is to exclude the existence of vascular iatrogenic lesions [28]. In most reported studies, pituicytomas show inert growth and have a long recurrence cycle, and patients with STR are more likely to relapse [7, 20, 29]. However, cases of recurrence after GTR have still been reported [29, 30]. In our study, we also confirmed the effect of the tumor resection extent on tumor progression. A retrospective analysis of previous patients found that sex is also one of the potential factors that affect tumor progression, but further verification is needed. Some authors suggest that stereotactic segmentation or conventional radiotherapy should be used systematically to control the residual tumor following STR of tumors [31], while others suggest that these treatments are limited to smaller tumors that do not affect visual acuity [32], but further data are needed to prove the effect. Recently, Mende et al. [12] indicated strong immunostaining for vascular endothelial growth factor receptor (VEGF-R) in a small series of patients with pituicytomas. Based on these results, anti-VEGF-R therapy (bevacizumab) and somatostatin analogs may be promising treatment options, although their use has not yet been approved.

## Conclusions

In summary, pituicytoma is exceedingly rare and is easily misdiagnosed as another type of tumor in the sellar region, such as pituitary adenoma and craniopharyngioma. Currently, GTR of the tumor is the most effective treatment. Pituicytoma has no specific radiological features, and the diagnosis must be pathologically confirmed. The blood supply of the tumor is very abundant. Methods to reduce intraoperative bleeding and subsequently increase the total resection rate of pituicytoma should be studied in the future. However, as we emphasized above, an accurate diagnosis of a suspicious lesion is an extremely difficult task.

## Supplementary Information

**Additional file 1: Table S1.** 68 cases of pituicytoma reported in the literature.

## Data Availability

Not applicable.

## Abbreviations

ETS: Endoscopic transsphenoidal surgery
EMA: Endomysial antibody
GFAP: Glial fibrillary acidic protein
GTR: Gross total resection
MRI: Magnetic resonance imaging
PFS: Progression-free survival
STR: Subtotal resection
SYN: Synaptophysin
TTF-1: Thyroid transcription factor-1
VEGF-R: Vascular endothelial growth factor receptor
