# Supplementary material for: Clinical features, radiological profiles, pathological features and surgical outcomes of pituicytomas: a report of 11 cases and a pooled analysis of individual patient data
Source: Mil Med Res. 2021 Jul 2;8:39. doi: 10.1186/s40779-021-00332-5 (PMC8252196; doi:10.1186/s40779-021-00332-5)
Supplement: Supplementary file 1 — Additional file 1: Table S1. 68 cases of pituicytoma reported in the literature. [file 40779_2021_332_MOESM1_ESM.docx]

**Supplementary table 1** 68 cases of pituicytoma reported in the literature

| Author | No. of cases | Year of publication |
| --- | --- | --- |
| Hammoud *et al* [1] | 10 | 2010 |
| Tian *et al* [2] | 1 | 2013 |
| Furtado *et al* [3] | 1 | 2010 |
| Brandão *et al* [4] | 1 | 2010 |
| Grote *et al* [5] | 1 | 2010 |
| Chu *et al* [6] | 1 | 2011 |
| Mao *et al* [7] | 2 | 2011 |
| Yang *et al* [8] | 3 | 2016 |
| Mende *et al* [9] | 9 | 2017 |
| Cambiaso *et al* [10] | 1 | 2015 |
| Richer *et al* [11] | 1 | 2016 |
| Guo *et al* [12] | 1 | 2016 |
| Neidert *et al* [13] | 1 | 2016 |
| Koutourousiou *et al* [14] | 3 | 2013 |
| Feng *et al* [15] | 3 | 2014 |
| Wang *et al* [16] | 11 | 2016 |
| Shenoy *et al* [17] | 1 | 2013 |
| Schmalisch *et al* [18] | 1 | 2012 |
| Secci *et al* [19] | 3 | 2012 |
| Yılmaz *et al* [20] | 1 | 2012 |
| Chakraborti *et al* [21] | 3 | 2013 |
| Zygourakis *et al* [22] | 5 | 2015 |
| Peron *et al* [23] | 1 | 2017 |
| Zunarelli *et al* [24] | 1 | 2011 |
| Zhang *et al* [25] | 1 | 2010 |
| Karamchandani *et al* [26] | 1 | 2012 |

1. Hammoud DA, Munter FM, Brat DJ, Pomper MG. Magnetic resonance imaging features of pituicytomas: analysis of 10 cases. J Comput Assist Tomogr. 2010;34(5):757-61.

2. Tian Y, Yue S, Jia G, Zhang Y. Childhood giant pituicytoma: a report and review of the literature. Clin Neurol Neurosurg. 2013;115(10):1943-50.

3. Furtado SV, Ghosal N, Venkatesh PK, Gupta K, Hegde AS. Diagnostic and clinical implications of pituicytoma. J Clin Neurosci. 2010;17(7):938-43.

4. Brandão RACS, Braga MHV, De Souza AA, Reis BL, de Lima FBF. Pituicytoma. Surg Neurol Int. 2010;1:79.

5. Grote A, Kovacs A, Clusmann H, Becker AJ, Niehusmann P. Incidental pituicytoma after accidental head trauma-case report and review of literature. Clin Neuropathol. 2010;29(3):127-33.

6. Chu J, Yang Z, Meng Q, Yang J. Pituicytoma: case report and literature review. Br J Radiol. 2011;84(999):e55-7.

7. Mao Z, Xiao W, Wang H, Li Z, Huang Q, He D, *et al*. Pituicytoma: Report of two cases. Oncol Lett. 2011;2(1):37-41.

8. Yang X, Liu X, Li W, Chen D. Pituicytoma: a report of three cases and literature review. Oncol Lett. 2016;12(5):3417-22.

9. Mende KC, Matschke J, Burkhardt T, Saeger W, Buslei R, Buchfelder M, *et al*. Pituicytoma-An outlook on possible targeted therapies. CNS Neurosci Ther. 2017;23(7):620-6.

10. Cambiaso P, Amodio D, Procaccini E, Longo D, Galassi S, Camassei FD, *et al*. Pituicytoma and Cushing’s disease in a 7-year-old girl: a mere coincidence? Pediatrics. 2015;136(6):e1632-6.

11. Richer M, Keith J. Coexisting pituicytoma and pituitary adenoma: a second coincidence? Hum Pathol. 2016;55:204-5.

12. Guo X, Fu H, Kong X, Gao L, Wang W, Ma W, *et al.* Pituicytoma coexisting with corticotroph hyperplasia: literature review with one case report. Medicine (Baltimore). 2016;95(10):e3062.

13. Neidert MC, Leske H, Burkhardt JK, Kollias SS, Capper D, Schrimpf D, *et al.* Synchronous pituitary adenoma and pituicytoma. Hum Pathol. 2016;47(1):138-43.

14. Koutourousiou M, Gardner P, Kofler J, Fernandez-Miranda J, Snyderman C, Lunsford L. Rare infundibular tumors: clinical presentation, imaging findings, and the role of endoscopic endonasal surgery in their management. J Neurol Surg B Skull Base. 2013;74(1):1-11.

15. Feng M, Carmichael JD, Bonert V, Bannykh S, Mamelak AN. Surgical management of pituicytomas: case series and comprehensive literature review. Pituitary. 2014;17(5):399-413.

16. Wang J, Liu Z, Du J, Cui Y, Fang J, Xu L, *et al*. The clinicopathological features of pituicytoma and the differential diagnosis of sellar glioma. Neuropathology. 2016;36(5):432-40.

17. Shenoy AS, Desai HM, Mehta JK. Pituicytoma: a case report with literature revisited. Indian J Pathol Microbiol. 2013;56(2):180-1.

18. Schmalisch K, Schittenhelm J, Ebner FH, Beuschlein F, Honegger J, Beschorner R. Pituicytoma in a patient with Cushing’s disease: case report and review of the literature. Pituitary. 2012;15(Suppl 1):S10-6.

19. Secci F, Merciadri P, Rossi DC, D’andrea A, Zona G. Pituicytomas: radiological findings, clinical behavior and surgical management. Acta Neurochir (Wien). 2012;154(4):649-57.

20. Yılmaz Ö, Turan A, Yiğit H, Duymuş M, Koşar U. Case of pituicytoma in childhood. Childs Nerv Syst. 2012;28(1):11-2.

21. Chakraborti S, Mahadevan A, Govindan A, Sridhar K, Mohan NSV, Satish IR, *et al.* Pituicytoma: report of three cases with review of literature. Pathol Res Pract. 2013;209(1):52-8.

22. Zygourakis CC, Rolston JD, Lee HS, Partow C, Kunwar S, Aghi MK. Pituicytomas and spindle cell oncocytomas: modern case series from the University of California, San Francisco. Pituitary. 2015;18(1):150-8.

23. Peron S, Mandelli J, Galante N, Colombo S, Locatelli D. Recurrent pituicytoma with pseudoaneurysm: report of a challenging case. World Neurosurg. 2017;105:1043.e1-e5.

24. Zunarelli E, Casaretta GL, Rusev B, Lupi M. Pituicytoma with atypical histological features: are they predictive of unfavourable clinical course? Pathology. 2011;43(4):389-94.

25. Zhang F, Chen J, You C. Pituicytoma: case report and review of the literature. Neurol India. 2010;58(5):799-801.

26. Karamchandani J, Syro LV, Uribe H, Horvath E, Kovacs K. Pituicytoma of the neurohypophysis: analysis of cell proliferation biomarkers. Can J Neurol Sci. 2012;39(6):835-7.
